# Neural bases of suicidal ideation and depression in young college students

**DOI:** 10.3389/fpsyg.2023.1141591

**Published:** 2023-02-21

**Authors:** Enrique López-Ramírez, Alma Dolores Pérez-Santiago, Marco Antonio Sánchez-Medina, Diana Matías-Pérez, Iván Antonio García-Montalvo

**Affiliations:** ^1^Division of Graduate Studies and Research, Tecnológico Nacional de México/Instituto Tecnológico de Oaxaca, Oaxaca, Mexico; ^2^Department of Chemical Engineering, Tecnológico Nacional de México/Instituto Tecnológico de Oaxaca, Oaxaca, Mexico

**Keywords:** suicidal ideation, depression, neural bases, cognitive flexibility, stress

## 1. Introduction

The risk factors that influence suicidal ideation are diverse, yet little has been achieved in the effective prediction of suicide (Fazakas-DeHoog et al., 2017; McManimen and Wong, 2020; MacPherson et al., 2022b). Recent studies have shown that patients with suicidal ideation (SI) or suicide attempt (SA) are referred to hospitals for treatment with drugs such as ketamine or clozapine as the only rehabilitation option. Despite all the studies conducted to reduce SI and SA through these drugs, the results have shown short-term effects. However, they are still considered the gold standard in the treatment of SI. Studies that have used the cognitive therapy-based randomized control trial approach have shown effectiveness, but to date there are still very few and many more longitudinal studies are needed to demonstrate that these patients have not had subsequent SA.

It is important to look for alternative treatments that promote a significant change in the increase in dopaminergic activity and the reward system. For example, the use of psilocybin, a natural substance derived from hallucinogenic mushrooms, has been shown to cause changes in neurogenesis, neuroplasticity, and brain regeneration, specifically cognitive flexibility (Strumila et al., 2021). From our perspective, we believe that it is necessary to abandon the idea that all those who suffer from SI or SA are mentally ill, which causes them to be stigmatized and subjected to psychiatric treatment, when what they need is a different vision of life as well as adaptive strategies to employ a new philosophy. Public policies should consider implementing programs that reinforce cognitive flexibility in addition to character strengths.

## 2. Cognitive flexibility, passive suicidal ideation, depression, and active suicidal ideation

Suicide and depression are among the most alarming phenomena in the world. The prevalence of depression and SI in the general population is considerable (Raj et al., 2019). More than 800,000 people die by suicide each year and 60% of those who have decided to take their own lives have been reported to suffer from major depressive disorder (Malhi et al., 2020). SI is considered a warning symptom and perhaps the primary predictor of suicide in patients with major depression. Passive suicidal ideation (PSI) may be an especially important early stage of the risk of suicide in older adults. While active SI refers to thoughts of suicide with or without intent to attempt and/or with a plan, PSI generally refers to a desire to die without thoughts of actual intent and without a plan to generate harm to oneself (Jordan et al., 2020). Although several regions of the brain related to SI and major depression have been identified in the scientific literature, knowledge based on the structural network of the brain and the relationship between these two constructs is still very limited (Scott, 1962; Liu et al., 2021).

Individuals with SI or SA have been found to exhibit a variety of cognitive vulnerabilities, including some deficits that have emerged as possible mechanisms underlying suicidal thinking and behavior (Geurts et al., 2009; Fazakas-DeHoog et al., 2017). Deficits in cognitive flexibility predict SI in clinical samples (MacPherson et al., 2022a). Malhi et al. (2019) conducted a study to analyze the neural basis of cognitive flexibility in the context of emotion in patients with depressed mood disorder using the Emotional Face-Word Stroop task and analyzing whether this impairment contributes to suicidal activity. Their results showed that cognitive flexibility was impaired in patients and that it was not modulated by the nature of the emotion; their findings suggest that cognitive performance is different between normal subjects and patients with SI and mood disturbances.

MacPherson et al. (2022b) demonstrated that subjects who have had SA have deficits in cognitive flexibility and greater impulsivity compared to normal subjects. Cognitive flexibility has been shown to mediate rumination and hopelessness, increasing vulnerability to SI (Ram et al., 2019). The underlying mechanism of suicide remains unclear and there is no animal model with endophenotypes implicated in suicide (Teng et al., 2022). The genetic basis of SI risk is poorly studied, especially in general population cohorts (Ahrens et al., 2022). The diathesis-stress model suggests that suicide is the result of an interaction between genetic vulnerability and environmental stressors (Stacy and Schulkin, 2022).

Suicidal thinking is related to functional alterations in brain circuits (Malhi et al., 2020). The study of neural correlates is considered of vital importance to understand the neurobiology of SI and for the development and implementation of targeted treatments (Ballard et al., 2015). It has been shown that alterations in the ventral extended PFC system can enhance suicidal thinking, given the preponderant role in negative self-referential thinking and rumination, in addition, suicidal thinking can be exacerbated by the dorsolateral prefrontal cortex, inferior frontal gyrus, rostromedial prefrontal cortex, and dorsal anterior cingulate cortex; it has also been shown that these alterations influence affective cognitive control, flexibility, and decision making (Kim et al., 2011; Auerbach et al., 2021).

Studies that have addressed suicide risk approaches from a genomic and clinical perspective have identified several biomarkers of SI involved in neuronal connectivity and activity, immune and inflammatory response, and inhibition of mammalian target rapamycin (mTOR) signaling pathways (De Berardis et al., 2018). Likewise, it has been reported that the amygdala and sgACC are associated with suicidal behavior, however, in a study conducted by Ballard and Zarate (2020), to test this relationship, they conducted a study with patients with major depression and found no relationship of these two regions with current suicidal thoughts. Executive functions are associated with the prefrontal cortex; for their study in the laboratory, tasks of cognitive control, working memory, and emotional decision making are used (Eduardo et al., 2017).

Alterations in cognitive flexibility are related to SI and behavior (Baeken et al., 2019). Cognitive flexibility can be understood in multiple ways, it can be interpreted as a cognitive ability or as a property of various cognitive processes (Ionescu, 2012). Typically, when a person switches from one task to another, there is a decrease in performance, but this is not the case when a person is focused on the same task (Murphy and Shin, 2022). Individuals with cognitive flexibility have greater confidence in their own abilities to behave effectively (Martin and Anderson, 1998; Martin et al., 2011).

Cognitive flexibility is the ability to mentally switch between different thoughts and action plans (Rikhye et al., 2018). This ability has been widely recognized as a basic function of cognitive control (Braem and Egner, 2018). It is a construct that is considered an emerging feature of executive functions whose measurement is typically performed in laboratories with task-switching neurological tasks (Dajani and Uddin, 2015). It has been defined in a variety of ways, some of which include at least one of the following three factors: the ability to adapt to change; the ability to think in a variety of categories and concepts; and the ability to perceive multiple perspectives or thoughts (Moore, 2013). It also refers to the ability to switch between a set of responses and process multiple sources of information simultaneously (Clearfield and Niman, 2012).

McManimen and Wong (2020) conducted a study to analyze the interaction of three aspects of executive functions that may have an impact on SI: cognitive flexibility, task switching, and distractibility. Cognitive rigidity has been associated with multiple adverse mental health problems, including SI (Novak et al., 2022). Novak et al. (2022) conducted a study with 40 psychiatric patients at risk of suicide; their results showed that cognitive flexibility was significantly associated with the most critical stage of SI.

## 3. Suicide from a psychosocial perspective

This diathesis-stress model suggests that cognitively inflexible individuals are unable to generate effective solutions when faced with stressful situations (Miranda et al., 2012). Vuong proposes the term near-suicide, which refers to the decision of a patient diagnosed with a severe mental illness to abandon treatment because it is too costly and represents damage to the family's finances. From this approach, the authors argue that patients may seek other means of recovery (traditional medicine, miracle cures, or fate). It can be considered as an indicated moment for them in which they are willing to undergo all kinds of experiences that lead them to face difficulties in a rational way. Cognitive flexibility would allow us to leave aside the beliefs of abandonment, detachment, desolation, and change them for other types of virtuous practices such as hope, forgiveness, empathy, among others proposed by positive psychology (Vuong et al., 2021, 2022a).

The desire to die may be consistent with the person's belief system, which is conditioned by circumstances, leading to feelings of abandonment, disillusionment, and anger (Zürcher, 2022); in this sense, most of the people in these conditions have surely tried to seek psychological help and still fail to improve their mental health; apparently, they are unable to change their perspective and their attitude toward life and the problems they face. We emphasize that more empirical evidence must be sought to demonstrate that it is possible to influence mindset with people who have reported deficits in cognitive flexibility and SI. In such a way, as Zürcher argues no one wants to end their life, what they want is to end their feeling of misery.

## 4. The mindsponge model

Vuong et al. (2022b), defines The Mindsponge as a mechanism that illustrates how a person can absorb new values and eject waning values conditionally based on contexts. In the case of people with SI, it is inevitable to think that their environment seems hostile to them and consequently they cannot attribute a high value or benefit to staying alive. From the mindsponge approach, the mindset can influence people's perception, attitudes, and beliefs, creating a self-protection mechanism for their self when their self-integrity is threatened (Vuong et al., 2022a). This may be one of the reasons why people with SI refuse or do not comply with to treatment. Therapies can be seen as cold and outside their own context in which someone who has not been through the same situation cannot understand what it really feels like to wish to end your life.

People with SI or SA can be considered mentally ill, as substance abusers can be characterized as mentally ill. There is ample evidence that their brain mechanisms are different from those of subjects without this condition in relation to the nucleus accumbens, amygdala, frontal cortex, and hippocampus (Koob and Volkow, 2010). Perhaps a person may have SI or SA, have deficits in the reward system or some other brain system, but should not be considered as a mentally ill patient to be controlled or disabled to protect them (Szasz, 2019). However, substance addicts have been able to rehabilitate themselves in self-help groups where they receive support from people who have also suffered from addiction and have found a purpose in life. Similarly, people with SI may find the sense of connectedness (Nguyen et al., 2021) and understanding they need.

The same authors argue that the idea of ending their life is only one option they can choose from based on what they are judging as cost-benefit, but there are others, such as seeking help, solving the problems, and doing more meaningful activities. We argue that these choices must be accompanied by significant people with whom they feel a deep connection and with whom they can begin to practice their newfound values.

## 5. Conclusions

In conclusion, we can say that suicide is a multi-causal and multi-dimensional phenomenon, in which a multitude of factors (sociological, psychiatric, and psychological) are involved. Two different profiles can be defined between people who commit suicide and those who attempt it. The profile of the completed suicide would be that of a male, older person, who had planned the act, who had chosen a method with high lethality, who lived an intrapersonal conflict, with low possibility of rescue, who was exposed to risk factors for a long time, which had few warning signs, few suicidal antecedents, or a low level of hostility.

From a psychosocial point of view, SI is not always related to other serious behavioral disorders. In the scientific literature, the wish to die prevails. From our perspective, no person has a desire to end his or her life, what is desired is not to continue living in the same way, and, not finding coping mechanisms, the only option they have is to end their suffering through death. More scientific evidence is needed to demonstrate that it is possible to transform the maladaptive schemas of people with SI into functional schemas through the promotion of resilience and wellbeing.

People with SI should not be seen as patients or pathological patients; they are human beings who are desolate and who feel they cannot adapt to the world. In this sense, we could argue that it is mentally healthy people who would need interventions to be a protective factor for people with SI. From our point of view, if they are willing to live through the torment prior to suicide, they would be in an ideal moment so that with peer-to-peer social support they can experience the desires to continue living, they need to revalue themselves and find a new purpose in life. In this sense, hope is a character strength that has helped to achieve subjective wellbeing and develop resilience in people with vulnerable mental health conditions.

It has not been possible to move fully from the diagnostic approach to the human perspective, i.e., to look at the sufferer as someone who needs someone significant to lean on. The diagnosis labels, disables, and isolates human beings from social life and makes them feel different from others. It is in the interest of large drug companies to continue the policy of medication as a mechanism to prevent suicide, but not to allow human flourishing. Both people with SI and people with apparent social functioning need to understand how to approach each other.

There are many people who would like to end their lives, some only numb the pain with opioids, methamphetamine, cocaine, and some others find other mechanisms or forms of dependence. It is important to stop focusing on the “disease” (drug use, suicide, gambling) and to find mechanisms for them to develop their strengths. We believe that, if there is social connection and hope as a strength of character, it is possible to move from cognitive inflexibility to cognitive flexibility, that is, from the modification of dysfunctional schemes to adaptive schemes to face the difficulties that arise.

For SA, the individual is more likely to be young women, through an impulsive act, with a low lethality method, also living conflicts at the interpersonal level, with high possibility of rescue, conjunctural precipitating factors, many warning signs, with previous attempts, high level of hostility, and greater psychopathology of character. Most explanatory models on suicidal behavior that take into account the interaction between risk factors is usually based on the diathesis-stress model, the individual with a certain biological predisposition (diathesis) is more vulnerable, when he has to face stressful life events, that is where he manifests suicidal behavior as he does not contemplate any other solution or alternative.

## Author contributions

EL-R, AP-S, MS-M, DM-P, and IG-M participated in the study concept, design, drafting, and critical review of the manuscript. All authors contributed to the article and approved the submitted version.
